# Recurrent Ischemic Stroke Despite Dabigatran Therapy: Unexpectedly Low Anti-IIa Activity Revealing Pharmacokinetic Treatment Failure

**DOI:** 10.7759/cureus.116768

**Published:** 2026-09-23

**Authors:** Nouhaila Bentama, Tagueniti Jalal, Said Hamdan, Brahim Meftout

**Affiliations:** 1 Cardiology, Ibn Sina Hospital, Mohammed V University, Rabat, MAR; 2 Cardiology, South Francilien Hospital Center, Corbeil-Essonnes, FRA; 3 Cardiology, Centre Hospitalier Sud Francilien, Corbeil-Essonnes, FRA

**Keywords:** anti-iia, apixaban, dabigatran, direct oral anticoagulant, paroxysmal atrial fibrillation, patent foramen ovale, pharmacokinetic treatment failure, recurrent stroke

## Abstract

Direct oral anticoagulants (DOACs) are the preferred therapy for stroke prevention in atrial fibrillation, but rare cases of pharmacokinetic resistance have been described, and objective biological confirmation remains scarce in the literature. We report the case of a man who experienced three ischemic strokes between the ages of 57 and 60 over a four-year period. The first episode, of cryptogenic origin, was attributed to a patent foramen ovale (PFO) associated with an interatrial septal aneurysm, leading to percutaneous closure. The recurrence led to a diagnosis of paroxysmal atrial fibrillation via an implantable cardiac monitor. Despite treatment with dabigatran 150 mg twice daily, a third stroke occurred. Pharmacokinetic treatment failure was documented by anti-IIa chromogenic testing, which had dropped to a residual level below 15 ng/mL despite supervised administration, adequate renal function, and no identified drug interactions. Switching to apixaban resulted in satisfactory on-therapy levels from Day 3. This case illustrates the value of prolonged rhythm monitoring after PFO closure and demonstrates apparent pharmacokinetic treatment failure with dabigatran, objectively confirmed by anti-IIa activity testing. Pharmacokinetically guided DOAC switching should be considered when recurrent ischemic events occur despite apparently adequate anticoagulation.

## Introduction

Paroxysmal atrial fibrillation (PAF) is one of the leading causes of cardioembolic stroke, accounting for approximately 15% to 20% of ischemic strokes [1]. Dabigatran, a direct thrombin inhibitor, demonstrated superiority over warfarin in preventing stroke in nonvalvular atrial fibrillation in the Randomized Evaluation of Long-Term Anticoagulation Therapy (RE-LY) trial [2]. However, rare cases of clinical and pharmacokinetic treatment failure with dabigatran have been described in the literature, raising the issue of drug-level monitoring of direct oral anticoagulants (DOACs) [3]. Furthermore, percutaneous patent foramen ovale (PFO) closure is recommended in young patients with cryptogenic stroke, but it is associated with a significant risk of post-procedural atrial fibrillation, which can itself become a source of embolic recurrence [4]. We report a clinical case illustrating these three situations in succession: stroke due to PFO, delayed diagnosis of atrial fibrillation, and subsequently confirmed apparent treatment failure with dabigatran, demonstrated by anti-IIa activity testing.

## Case presentation

A 57-year-old man with no cardiovascular risk factors presented on Day 1 with a transient left sensorimotor deficit. Brain MRI revealed a right superficial sylvian infarction (Figure 1A) with a 5 mm cortical thrombus (susceptibility-weighted angiography (SWAN) sequence). Transoesophageal echocardiography confirmed a high-risk PFO associated with an atrial septal aneurysm (excursion 19 mm, more than 20 bubbles on Valsalva manoeuvre). Percutaneous closure was performed two months later (Month 2) using a 25 mm AMPLATZER PFO Occluder (Abbott, Abbott Park, Illinois, USA), followed by dual antiplatelet therapy for three months, then aspirin alone. Follow-up echocardiography at three months and one year after closure confirmed complete device sealing with negative bubble tests (Figure 2).

**Figure 1:**
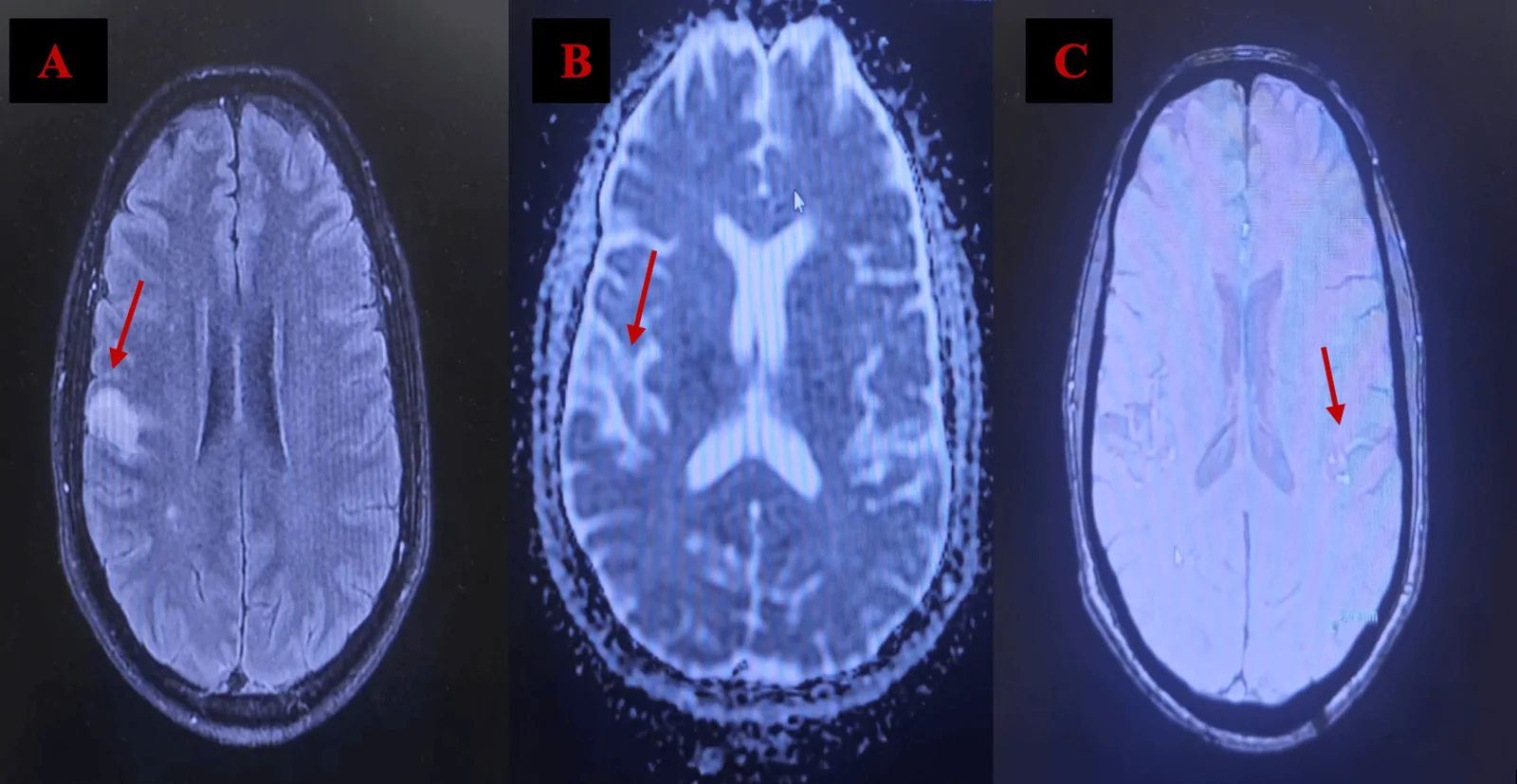
Axial brain MRI at each of the three ischemic events, with the corresponding lesion indicated by a red arrow. (A) Day 1: fluid-attenuated inversion recovery (FLAIR) sequence showing a right superficial sylvian cortical hyperintensity, consistent with the first infarction. (B) Month 32: apparent diffusion coefficient (ADC) map showing restricted diffusion in the right posterior sylvian territory, consistent with the second infarction. (C) Month 47: susceptibility-weighted angiography (SWAN) sequence showing the thrombus at the time of the third event.

**Figure 2:**
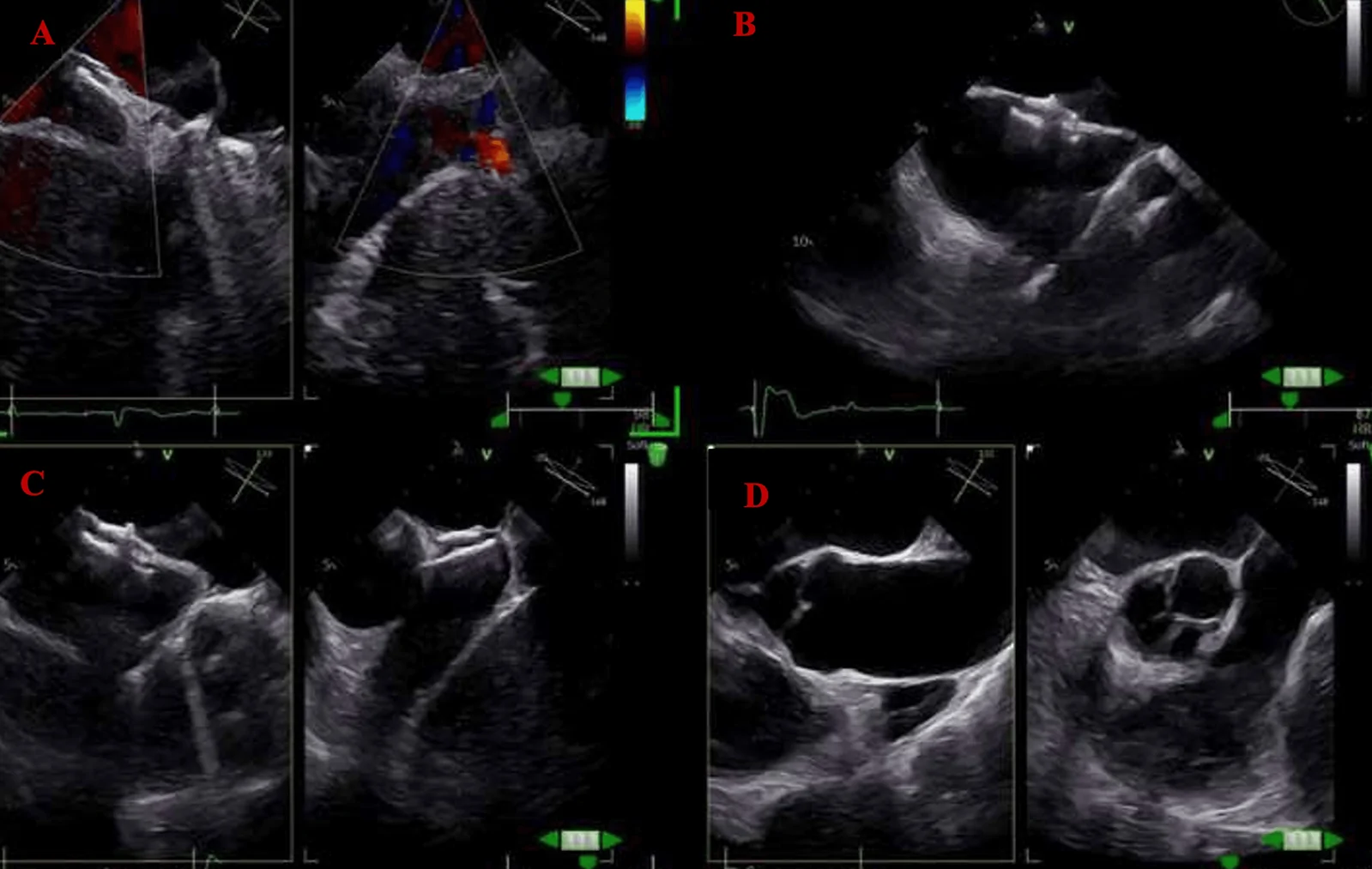
Transoesophageal echocardiography showing the AMPLATZER PFO occluder in place, with complete sealing on colour Doppler and negative bubble tests. (A) Two-dimensional views with colour Doppler, showing no residual trans-septal shunt. (B, C, D) Two-dimensional views of the interatrial septum and the AMPLATZER occluder device, confirming complete apposition and septal sealing.

Approximately three years after the initial presentation, the patient experienced a second transient neurological episode: left facial palsy and dysarthria resolving within two hours. MRI demonstrated a new right posterior sylvian infarction (Figure 1B) with a 6 mm M3 thrombus (SWAN), confirmed by time-of-flight magnetic resonance angiography (TOF-MRA) (Figure 1B) . Transcranial Doppler bubble test was negative, ruling out device failure. In-hospital telemetry during this admission did not reveal any arrhythmia, prompting the decision to proceed to extended rhythm monitoring. Given the distal nature of the occlusion and the minor deficit (National Institutes of Health Stroke Scale (NIHSS) score of 1) [5], thrombolysis was not indicated. An implantable cardiac monitor (Reveal LINQ, Medtronic, Minneapolis, Minnesota, USA) was implanted one month later, and dabigatran 150 mg twice daily was initiated. Subsequent transoesophageal echocardiography performed two months after this second stroke confirmed device integrity with no atrial thrombus. Monitor interrogation five months after the second stroke revealed multiple paroxysmal atrial fibrillation episodes lasting several hours, confirming a cardioembolic aetiology.

Ten months later, the patient, now 60 years old, presented a third episode: isolated speech arrest resolving after 30 minutes followed by persistent mild word-finding difficulty and phonemic paraphasias. MRI showed a 7 mm left M3/M4 thrombus (SWAN), hypoperfusion with arterial transit artefacts (arterial spin labelling), and slow flow on fluid-attenuated inversion recovery (FLAIR), without restricted diffusion (Figure 1C). Real-time monitor interrogation confirmed atrial fibrillation on the day of admission. Intravenous thrombolysis with tenecteplase (0.25 mg/kg) was administered on the basis of standard clinical and radiological criteria (persistent language disturbance despite resolution of the initial speech arrest; MRI findings excluding haemorrhage), without awaiting coagulation panel results. The thrombin time subsequently returned prolonged (33.0 s versus a control of 16.0 s), consistent with a measurable residual dabigatran effect; retrospectively, this reinforces the finding of pharmacokinetic treatment failure rather than complete drug clearance. Dabigatran reversal with idarucizumab was not administered. Thrombolysis resulted in complete neurological recovery at one hour (NIHSS score of 0).

The critical finding was the anti-IIa chromogenic assay, performed using dabigatran-specific calibrators in accordance with standard laboratory practice: the trough level on admission (more than 12 hours post-dose) was below 15 ng/mL, far below the usual on-therapy range for dabigatran 150 mg twice daily (33-151 ng/mL). The peak level after supervised in-hospital administration reached 106 ng/mL, suggesting adequate absorption but reduced dabigatran exposure. Adherence was verified by nursing staff during hospitalisation, and the patient also reported no missed doses in the outpatient period preceding this admission. Renal function was adequate for standard dosing (creatinine clearance: 65 mL/min), and no drug interactions were identified. Given this apparent pharmacokinetic treatment failure, dabigatran was switched to apixaban 5 mg twice daily. Three days after the switch, anti-Xa levels showed measurable on-therapy exposure to apixaban, with a trough of 68 ng/mL (within the expected range) and a peak of 124 ng/mL (slightly below the expected range). The patient was discharged asymptomatic (modified Rankin Scale (mRS) score of 0) [6]. Relevant laboratory findings across the three hospitalisations are summarised in Table 1, and the clinical timeline is summarised in Table 2.

**Table 1:** Relevant laboratory findings across the three hospitalisation periods. PT: prothrombin time; aPTT: activated partial thromboplastin time; hs: high-sensitivity; NT-proBNP: N-terminal pro-B-type natriuretic peptide; LDL: low-density lipoprotein; HbA1c: glycated haemoglobin (haemoglobin A1c).

Parameter	2nd Admission	3rd Admission	3rd Admission during stay	Reference range
Coagulation and anticoagulation monitoring				
PT (%)	83	87	76	70%-130%
aPTT ratio	0.82	0.81	0.88	< 1.18
Thrombin time (s) Patient / Control	17.8 / 16.4	33.0 / 16.0	24.3 / 16.0	≈ Control
D-dimers (ng/mL)	550	< 270	900	50-420
Fibrinogen (g/L)	2.8	3.3	3.6	1.9-4.3
Anti-IIa activity (Dabigatran, ng/mL)	Not measured	< 15 (trough-subtherapeutic)	106 (peak-low normal)	Trough: 33-151; Peak: 76-285
Anti-Xa activity (Apixaban, ng/mL)	—	—	Peak: 124; Trough: 68	Trough: 58-206 / Peak: 132-343
Cardiac biomarkers				
Troponin T hs (pg/mL)	10.73	8.19	10.70	< 14
NT-proBNP (pg/mL)	193	228	91	< 920
Renal function				
Creatinine (µmol/L)	84	90	88	59-104
CrCl, Cockcroft (mL/min)	~68	65	—	> 50
Metabolic and lipid profile				
LDL cholesterol (g/L)	0.74	1.49	—	< 1.00
HbA1c (%)	5.8	6.1	—	4.5-6.5

**Table 2:** Clinical timeline of the three recurrent ischemic strokes over four years, illustrating the sequential diagnoses of PFO-related embolism, paroxysmal atrial fibrillation, and apparent pharmacokinetic treatment failure with dabigatran confirmed by anti-IIa chromogenic assay, with successful switch to apixaban guided by therapeutic drug monitoring. Time points are expressed relative to the initial presentation. PFO: patent foramen ovale; ASA: atrial septal aneurysm; TEE: transoesophageal echocardiography; TTE: transthoracic echocardiography; DAPT: dual antiplatelet therapy; SWAN: susceptibility-weighted angiography; TOF-MRA: time-of-flight magnetic resonance angiography; MCA: middle cerebral artery; ASL: arterial spin labelling; AF: atrial fibrillation; PAF: paroxysmal atrial fibrillation; NIHSS: National Institutes of Health Stroke Scale; mRS: modified Rankin Scale; IV: intravenous; CrCl: creatinine clearance; FLAIR: fluid-attenuated inversion recovery.

Time point	Event	Clinical / paraclinical findings
Day 1	1st ischemic stroke, right sylvian territory	Sudden-onset left sensorimotor deficit, fully resolved. MRI: right superficial sylvian infarction, 5 mm cortical thrombus (SWAN sequence). TEE: large PFO + ASA (excursion 19 mm, >20 bubbles on Valsalva). No aortic atheroma.
Month 2	Percutaneous PFO closure	AMPLATZER PFO Occluder 25 mm (Abbott). DAPT × 3 months → aspirin alone. TTE at 3 months and 1 year after closure: complete device sealing, negative Valsalva bubble tests.
Month 32	2nd ischemic stroke, right sylvian territory	Left facial palsy and dysarthria, resolved in 2 hours. MRI: right posterior sylvian infarction, 6 mm M3 thrombus (SWAN), confirmed M3 occlusion (TOF-MRA). Transcranial Doppler bubble test negative, device intact. NIHSS = 1. No thrombolysis (distal occlusion, minor deficit). Dabigatran 150 mg twice daily initiated. Reveal LINQ implanted one month later (Month 33). TEE two months later (Month 34): device sealed, no atrial thrombus, negative bubble tests. CrCl: 65 mL/min.
Month 37	PAF confirmed by Reveal LINQ	Multiple paroxysmal AF episodes lasting several hours identified retrospectively between Month 34 and Month 37 (ventricular rate: 90-125 bpm). Aetiology revised: cardioembolic, paroxysmal atrial fibrillation. Dabigatran continued.
Month 47	3rd ischemic stroke on dabigatran, left sylvian territory	Isolated speech arrest, spontaneously resolved after 30 minutes. MRI: 7 mm left M3/M4 thrombus (SWAN), slow flow on FLAIR (5 slices), hypoperfusion with arterial transit artefacts (ASL). TOF-MRA: reduced distal left MCA branches. Reveal monitor: AF confirmed day of admission. IV thrombolysis with tenecteplase 0.25 mg/kg → complete recovery at 1 hour (NIHSS = 0). MRI at 24 hours: small left posterior sylvian ischaemic spot, no haemorrhagic transformation, branch recanalisation. Key finding: anti-IIa chromogenic assay: trough (>12 h post-dose) <15 ng/mL (usual on-therapy range: 33-151 ng/mL), confirming pharmacokinetic resistance to dabigatran; peak (supervised in-hospital dose) 106 ng/mL, indicating adequate drug exposure but a subtherapeutic residual level. Adherence verified; CrCl: 65 mL/min (adequate for standard dosing); no drug interactions identified.
Month 47, Day 3	Switch to apixaban (pharmacology-guided decision)	Apixaban 5 mg twice daily. Anti-Xa activity: peak 124 ng/mL (expected 132-343) and trough 68 ng/mL (expected 58-206). Discharged asymptomatic (mRS = 0, NIHSS = 0). Bisoprolol 2.5 mg/day and atorvastatin 80 mg/day.

## Discussion

This case illustrates three major clinical lessons that are sequential and interrelated. The first concerns the need for prolonged rhythm monitoring following percutaneous PFO closure. Several randomised trials have demonstrated that PFO closure significantly reduces the risk of recurrent cryptogenic stroke, but at the cost of an increased risk of post-procedural atrial fibrillation, particularly in the weeks following implantation [4]. A recent meta-analysis reported a pooled incidence of new-onset atrial fibrillation of approximately 5% after percutaneous closure, compared with under 2% with conservative management (odds ratio: 3.17) [7]. In our case, atrial fibrillation was detected only 2.5 years after closure, thanks to the implantable cardiac monitor, underscoring the importance of prolonged cardiac monitoring in this population. Without this monitoring, the cardioembolic aetiology would have remained undiagnosed, and anticoagulant therapy would not have been initiated.

The second, and most novel, finding concerns apparent pharmacokinetic treatment failure with dabigatran. The true incidence of pharmacokinetic treatment failure with DOACs is not well established, as systematic drug-level monitoring is not standard practice; while non-adherence remains the most commonly cited explanation for treatment failure with DOACs, our case stands out because it provides objective biological confirmation obtained under controlled conditions. The residual anti-IIa level plummeted (below 15 ng/mL, compared to a usual on-therapy range of 33 to 151 ng/mL), despite supervised administration, adequate renal function (clearance: 65 mL/min), and the absence of any identified drug interactions [8]. It is noteworthy that the peak level, at 106 ng/mL, at the lower limit of normal, did not result in an adequate residual level, suggesting reduced dabigatran exposure rather than a failure of absorption, although serial concentration measurements would be needed to formally confirm accelerated elimination and estimate the drug's half-life.

This pharmacokinetic profile is consistent with data in the literature. A prospective multicentre study found that patients with atrial fibrillation who suffered an embolic stroke while on dabigatran had significantly lower anti-IIa plasma levels than controls on the same therapy without adverse events, suggesting that subtherapeutic drug exposure is a genuine mechanism of treatment failure [9]. In the RE-LY trial, residual concentrations of dabigatran 150 mg twice daily showed interindividual variability of more than fivefold, with some patients reaching levels well below the usual on-therapy range despite confirmed dosing [10]. The underlying mechanisms are not fully understood. Dabigatran etexilate is a substrate of intestinal P-glycoprotein, encoded by the *ABCB1* gene. Polymorphisms in this gene, notably the rs4148738 variant, have been associated with reduced dabigatran trough concentrations, potentially due to increased intestinal efflux of the drug [11]. Furthermore, since approximately 80% of dabigatran is eliminated renally, interindividual variability in tubular secretion could also contribute to accelerated elimination, even in the presence of apparently normal renal function [12]. These data suggest that a genetic component, which was not investigated in our case, and which constitutes a limitation of this report, could explain the documented apparent pharmacokinetic treatment failure. A pharmacogenomic assessment including sequencing of the *ABCB1* and *CES1* genes should be considered in similar situations. Adherence was also confirmed for the outpatient period preceding the third event, with the patient reporting no missed doses on direct questioning.

The third key takeaway concerns the role of pharmacokinetic monitoring of DOACs in the management of recurrent strokes. Chromogenic anti-IIa activity testing is not recommended for routine use, but is an essential tool when treatment failure is suspected. The 2024 European Society of Cardiology (ESC) guidelines on atrial fibrillation specify that in the event of a recurrent stroke while on curative anticoagulation, a comprehensive diagnostic evaluation must be conducted as a priority, including verification of adherence, dosing, and drug interactions, before concluding that treatment has failed [13]. In our case, confirmation of measurable anti-Xa activity under apixaban as early as Day 3 after the switch, with a trough of 68 ng/mL within the usual range and a peak of 124 ng/mL slightly below the usual range [8], was consistent with adequate drug exposure and supported the therapeutic decision, underscoring the value of pharmacokinetically guided monitoring. Unlike dabigatran, apixaban is metabolised primarily by the liver (CYP3A4) and is not exclusively dependent on intestinal P-glycoprotein for its absorption, which may explain the more reassuring pharmacokinetic profile observed after the switch in our case. At the most recent clinical follow-up, the patient remained free of recurrent thromboembolic or bleeding events, although formal long-term outcome data are not yet available. A systematic approach to pharmacological monitoring of DOACs in cases of recurrent stroke under curative anticoagulation should be integrated into management algorithms, in accordance with emerging data from the literature.

## Conclusions

This case demonstrates the importance of prolonged rhythm monitoring following PFO closure in patients who have experienced a cryptogenic stroke, as well as the existence of apparent pharmacokinetic treatment failure with dabigatran, which can be suggested by measuring anti-IIa activity. In the event of a recurrent stroke while on therapeutic anticoagulation, pharmacokinetic testing should be considered when recurrent ischemic events occur despite apparently adequate anticoagulation. Switching to a DOAC from a different class, guided by pharmacokinetics, may represent a reasonable therapeutic strategy in this rare but potentially serious situation, although this observation is based on a single case and longer-term follow-up would be needed to confirm sustained clinical effectiveness.
